# Machine learning for risk stratification in the emergency department (MARS-ED) study protocol for a randomized controlled pilot trial on the implementation of a prediction model based on machine learning technology predicting 31-day mortality in the emergency department

**DOI:** 10.1186/s13049-024-01177-2

**Published:** 2024-01-23

**Authors:** Paul M.E.L. van Dam, William P.T.M. van Doorn, Floor van Gils, Lotte Sevenich, Lars Lambriks, Steven J.R. Meex, Jochen W.L. Cals, Patricia M. Stassen

**Affiliations:** 1 Department of Internal Medicine, Division of General Internal Medicine, Section Acute Medicine, Maastricht University Medical Center +, PO Box 5800, Maastricht, 6202 AZ The Netherlands; 2 Central Diagnostic Laboratory, Department of Clinical Chemistry, Maastricht University Medical Center +, Maastricht, The Netherlands; 3 Department of Family Medicine, Care and Public Health Research Institute (CAPHRI), Maastricht University, Maastricht, The Netherlands; 4 School for Cardiovascular Diseases (CARIM), Maastricht University, Maastricht, The Netherlands

**Keywords:** Machine learning, Artificial intelligence, Emergency department, Implementation, Risk stratification, Prediction, Mortality, Prognosis

## Abstract

**Background:**

Many prediction models have been developed to help identify emergency department (ED) patients at high risk of poor outcome. However, these models often underperform in clinical practice and their actual clinical impact has hardly ever been evaluated. We aim to perform a clinical trial to investigate the clinical impact of a prediction model based on machine learning (ML) technology.

**Methods:**

The study is a prospective, randomized, open-label, non-inferiority pilot clinical trial. We will investigate the clinical impact of a prediction model based on ML technology, the RISK^INDEX^, which has been developed to predict the risk of 31-day mortality based on the results of laboratory tests and demographic characteristics. In previous studies, the RISK^INDEX^ was shown to outperform internal medicine specialists and to have high discriminatory performance. Adults patients (18 years or older) will be recruited in the ED. All participants will be randomly assigned to the control group or the intervention group in a 1:1 ratio. Participants in the control group will receive care as usual in which the study team asks the attending physicians questions about their clinical intuition. Participants in the intervention group will also receive care as usual, but in addition to asking the clinical impression questions, the study team presents the RISK^INDEX^ to the attending physician in order to assess the extent to which clinical treatment is influenced by the results.

**Discussion:**

This pilot clinical trial investigates the clinical impact and implementation of an ML based prediction model in the ED. By assessing the clinical impact and prognostic accuracy of the RISK^INDEX^, this study aims to contribute valuable insights to optimize patient care and inform future research in the field of ML based clinical prediction models.

**Trial registration:**

ClinicalTrials.gov NCT05497830. Machine Learning for Risk Stratification in the Emergency Department (MARS-ED). Registered on August 11, 2022. URL: https://clinicaltrials.gov/study/NCT05497830.

**Supplementary Information:**

The online version contains supplementary material available at 10.1186/s13049-024-01177-2.

## Introduction

The accurate assessment of the severity of the patient’s disease is an important challenge for physicians in the emergency department (ED). As the number of patients who visit the ED increases worldwide, crowding increases pressure on ED physicians, affecting not only patient satisfaction, but also placing the patient at risk of delayed treatment, increased in-hospital length of stay and increased mortality [1–5]. Fast and reliable discrimination between high and low risk patients may assist in proper allocation and management of resources, improving acute healthcare for patients in the ED.

Stratification of ED patients into low and high risk of poor outcome has traditionally relied on the clinical intuition of ED physicians and nurses [6]. However, previous studies have shown that ED physicians experience a high cognitive load, having to make important clinical decisions for their patients, often based on incomplete data, while being interrupted regularly and attending several patients at the same time [7–9]. Several clinical prediction models, e.g. modified early warning score (MEWS), sepsis-related organ failure (SOFA) score, and risk stratification in the ED in acutely ill older patients (RISE UP), have been developed to aid clinical decision-making [10–12]. Since many models have not been externally validated, their discriminatory performance may be overestimated. Furthermore, implementation of these models may be difficult because some of them have been developed for a specific diagnosis, which may not yet have been established in the ED. Given the heterogeneity of ED patients, finding a single prediction model that covers the entire clinical spectrum remains difficult [13].

Recently, machine learning (ML) technology has emerged as a promising approach to develop new prediction models [14, 15]. One such new prediction model, the RISK^INDEX^, has been developed at the Maastricht University Medical Center (MUMC+) [16]. The RISK^INDEX^ utilizes sex, age and routine laboratory tests from the ED to predict 31-day mortality. The RISK^INDEX^ was externally validated in three other medical centers demonstrating an AUC of 0.88 to 0.98 ^16^. Although ML based prediction models show great potential, to our knowledge, randomized clinical trials to investigate their actual clinical impact have rarely been performed.

In this context, the MARS-ED study aims to evaluate the clinical impact of the RISK^INDEX^ in the ED. This pilot randomized study will assess both the magnitude of the clinical impact and the prognostic accuracy of the RISK^INDEX^ in a large study sample.

## Methods

### Study objectives and endpoints

The primary objective of this study is to assess the prognostic accuracy and the clinical impact of the RISK^INDEX^ prediction model. Secondary objectives of this study are to assess: (1) the number and type of changes in medical treatment after presentation of the RISK^INDEX^ to the ED physician; and (2) the prognostic accuracy of the RISK^INDEX^ as compared to that of the physician’s clinical intuition and that of other clinical prediction models (e.g. MEWS, SOFA, RISE UP score).

### Trial design and setting

The MARS-ED study is designed as an investigator-initiated, open-label, randomized, non-inferiority, pilot clinical trial. Adult patients presenting to the ED who are primarily assessed and treated by an internal medicine specialist will be randomized in a 1:1 ratio to the control group or the intervention group. This study is not blinded, since the physician needs to be informed of the RISK^INDEX^ in order to assess the size of the clinical impact of the RISK^INDEX^.

The study will be conducted in the ED of the MUMC+, which is a secondary/tertiary care medical center in the Netherlands, with 5,500 patients visiting the ED for assessment by an internal medicine specialist each year. In contrast to many other countries where patients can visit the ED without referral by a health care professional (open access ED), nearly all ED patients in the Netherlands after referred after an initial triage process by a general practitioner, a medical specialist or ambulance.

This study protocol is designed in accordance with the Standard Protocol Items: Recommendations for Interventional Trials - Artificial Intelligence (SPIRIT-AI) and the Consorted Standards of Reporting Trials (CONSORT) guidelines (Supplementary Table 1) [17, 18]. This study has been approved by the medical ethical committee (METC) of the MUMC + on June 21st 2022 (METC 21–068) and is registered at clinicaltrials.gov (NCT05497830). The study will be conducted according to the principles of the Declaration of Helsinki (version 2013, July 9th 2018) and in accordance with the standards of Good Clinical Practice. The study is expected to last from September 2022 to September 2024.

### Recruitment and selection of eligible patients

All adult ED patients (18 years or older) who are assessed and treated by internal medicine specialist or their residents and who meet the inclusion criteria will be asked informed consent to participate in this study by a member of the research team.

As patients may be unconscious, in a state of delirium or otherwise unable to provide informed consent, a legal representative may make the decision on participation as well. In case of absence of such representative, a deferred consent procedure may take place, in which consent is implied and randomization will take place according to the study protocol and the patient will receive medical care as usual [19]. Within 72 h after randomization, a member of the research team will contact the patient to ask for informed consent. If this is not possible, e.g. because the patient has already been discharged from the hospital before a member of the research team was able to contact the patient, the patient dies before giving informed consent, or the patient (or representative) remains unable (or unavailable) to give informed consent for more than 72 h after randomization, data of this patient will be deleted.

### Study intervention and procedure

When a patient enters the ED for assessment and treatment by an internal medicine specialist, the patient will immediately be assessed for eligibility and randomized as soon as informed consent is obtained. Obtaining informed consent is performed by a member of the research team and involves explaining the study intervention and procedure to the patient and handing out a patient information form. The inclusion criteria and exclusion criteria are shown in Table 1.

**Table 1:** Inclusion and exclusion criteria

Inclusion criteria	Exclusion criteria
Patients must meet all of the following criteria: - Adult (18 years or older) - Primarily assessed and treated by an internal medicine specialist in the ED - At least 4 laboratory test results available within the first 2 h of the ED visit - Willing to provide written informed consent (either directly or after deferred consent)	Patients who meet any of the following criteria: - Less than 4 laboratory test results available within the first 2 h of the ED visit - Unwilling to provide written informed consent

ED, emergency department

After inclusion, the patient is allocated to either the intervention group or the control group through randomization. An overview of the patient’s timeline is shown in Supplemental Fig. 1. Then, the patients will receive medical care in the ED as usual. After complete assessment of the patient, in both the intervention group and control group, the physicians will be asked questions regarding their clinical intuition (Table 2). In the intervention group, the RISK^INDEX^ will be presented to the physician together with the average mortality risk for ED patients with the same age and sex, and subsequently questions will be asked concerning agreement of their clinical intuition with the RISK^INDEX^ and whether the presented RISK^INDEX^ leads to changes in the treatment plan (Table 2). If the patient is admitted to the hospital, the attending physician on the medical ward will be asked the questions regarding clinical intuition on the first day of admission. A member of the research team immediately records the answers to the questions of all questionnaires in an electronic case record form, which was specially designed for this study.

**Table 2:** Questionnaires regarding clinical intuition and medical treatment changes

**Questionnaire regarding clinical intuition (both intervention group and control group)**
- Surprise question: “Would you be surprised if this patient dies within the next 31 days?” (yes/no) [20, 21] - Concern question: “How concerned are you about the health of this patient?” (Likert scale 1–10) [6] - Severity question: “How severely ill do you find this patient?” (Likert scale 1–10) [22] - “Do you think that this patient will be admitted to the hospital for more than 7 days?” (yes/no) - “Do you think that this patient will be admitted to the ICU?” (yes/no)
**Questionnaire regarding medical treatment changes (intervention group only)**
- “Is the RISK^INDEX^ higher than, equal to, or lower than you expected?” - “Do you want to alter the medical treatment plan based on the RISK^INDEX^?” (yes/no) - “If yes, which part of the treatment plan?“ (reassessment during current ED visit; order additional investigations and consultations; admission or discharge; consultation with an ICU specialist and/or admission to ICU; removing or emplacing treatment restrictions) ^a^

ED, emergency department; ICU, intensive care unit

^a^ The physician will be asked to specify which part of the treatment plan was altered by choosing any of the given options

### The RISK^INDEX^

The RISK^INDEX^ was developed using ML technology at the MUMC + and aims to predict all-cause 31-day mortality for adults ED patients [16]. The score is calibrated ranging from 0 to 100, where a high RISK^INDEX^ indicates a high risk of mortality, and a low RISK^INDEX^ indicates a low risk of mortality.

The RISK^INDEX^ is based on age, sex and the results of at least four routine laboratory tests in the ED performed within the first two hours of the ED visit. All laboratory values are used to calculate the RISK^INDEX^ with the exception of tests that are prevalent less than 0.01%. The most commonly ordered laboratory values are creatinine, complete blood count (CBC), sodium, potassium, C-reactive protein (CRP), blood urea nitrogen (BUN), glucose, alanine transaminase (ALAT), aspartate aminotransferase (ASAT), creatine kinase (CK), and platelets. A detailed prescription regarding the RISK^INDEX^ is shown in Supplemental data.

### Allocation and blinding

Patients will randomly be allocated in a 1:1 ratio to the intervention group or the control group (Supplemental Fig. 1). This randomization is automatically performed by a computer system using block randomization of 100 patients. Electronic randomization is performed automatically using an online program. The allocation is presented within 2 h after presentation of the patient to the ED. The study is an open-label study that is not blinded for physicians.

### Follow-up

After the patients’ visit to the ED, the follow-up regarding 31-day mortality and other endpoints will be checked by reviewing the medical records. In the Netherlands, all deaths are registered by the municipal administration office, and these data are linked to the medical records.

### Drop-out/withdrawal

Patients can leave the study at any time for any reason without any consequences if they wish to do so. The investigator can decide to withdraw a patient from the study for urgent medical reasons. If a patients withdraws from the study, the data up until that moment will be used in study analysis. Data of patients who are unwilling to participate in the study after a deferred consent procedure took place will be deleted and will not be used in the study analysis. The patient will not be replaced, as the study design takes less than 10% drop-out into account. Considering the short duration of the study follow-up, the expected drop-out rate is low.

### Adverse event monitoring

The presentation of the RISK^INDEX^ to the ED physician and the possibly resulting change in the medical treatment plan is considered to have a low risk of undesirable outcomes. The 31-day mortality is expected to remain around the usual average, which is approximately 10%. Possible complications will be closely monitored by the researchers and will be followed until they have abated, or until a stable situation has been reached.

### Data collection methods

The researcher will collect the results of laboratory tests, which are already routinely measured in the ED for internal medicine patients. The laboratory data, sex and age are used to determine the RISK^INDEX^. In addition, the physician will provide answers on the questions regarding their clinical intuition.

Patient characteristics will be retrieved from medical records and include: demographics (age, sex), comorbidities, mode of transportation to the ED (ambulance or own transport), reason for ED visit (according to the international classification of diseases (ICD-) 10 system) [23], date and time of the ED visit, and triage category. The following vital signs will be retrieved: heart rate (HR), blood pressure (BP), respiratory rate (RR), oxygen saturation, temperature, and Glasgow coma scale (GCS). Furthermore, we will collect data on hospital admission, admission to intensive care unit (ICU), treatment restrictions and all-cause mortality within 31 days after the ED visit. The data regarding patient characteristics, vital signs and outcomes will be retrieved manually. To ensure the quality of these data (i.e. to check whether the data are correct and complete), we will perform a double-check by another member of the research team and/or the study monitor. The retrieval of data regarding the results of laboratory tests and the results of the RISK^INDEX^ will be automated.

Data will be stored anonymously in an online electronic case record form in CASTOR, which is available for researchers in all participating study centers. Personal data will be handled in compliance with the EU General Data Protection Regulation and the Dutch Act on Implementation of the General Data Protection Regulation, as well as the “Algemene Verordening Gegevensbescherming)” (law on the protection of general data) [24, 25].

The patient identification code list will be stored digitally and encrypted with a strong password. The patient data and documents will be stored for 15 years. Data may be used for other studies which are in line with the current study, as approved by the METC. Data monitoring will be performed by the Clinical Trial Center Maastricht (CTCM).

### Sample size

This pilot trial has an explorative aim, providing future trials with estimates of effect sizes and potential clinical impact of the RISK^INDEX^. This study aims to provide robust estimates for the likely recruitment and retention rates and give an indication of the potential variability in the proposed outcome measures, which will in turn be used to inform the power calculation for a future definitive randomized controlled trial (RCT). We calculated a required sample size of 784 patients to detect a 2% difference in the number of changes in medical treatment after presentation of the RISK^INDEX^ between the control group and the intervention group with a power of 0.8.

Yearly, approximately 5,500 patients are treated by an internal medicine specialist at the ED of MUMC+. Considering fluctuations in the number of patients presenting to the ED and moments of crowding, there may be moments where there will not be enough time to include patients and/or to complete the questionnaires. Therefore, we expect a total sample size of 1300 patients during the inclusion period.

### Statistical analysis

All analyses will be performed using the SPSS software (IBM Corp. Released 2021. IBM SPSS Statistics for Windows, version 28.0. Armonk, NY: IBM Corp).

The prognostic accuracy of the RISK^INDEX^ and the physician’s clinical intuition to predict adverse outcomes will be analyzed using an area under the receiver operating characteristics curve (AUC). To assess the clinical impact of the RISK^INDEX^, the number of changes in medical treatment based on the RISK^INDEX^ will be calculated. The prognostic accuracy of the RISK^INDEX^ will also be compared to that of the physician’s clinical intuition and to that of other clinical prediction models. Sub-analyses will be performed on the ED physicians’ characteristics, i.e. level of experience and function in relation to the accuracy of their clinical intuition. Summary estimates of effects will be presented along with their 95% confidence intervals (CI). Differences between the intervention and control groups will be presented in the form of an unadjusted mean difference for continuous outcomes, and an odds ratio for binary outcomes. Exploratory analysis using ANCOVA for continuous variables and logistic regression for binary outcomes will consider adjustment for the stratification variables in assessment of the treatment effects. Baseline characteristics will be summarized for all patients, and separately for the intervention group and control group. Feasibility and process evaluation data, such as practice recruitment rate, implementation and uptake of and adherence to the intervention, and follow-up rates will be summarized and presented as percentages.

## Discussion

Rapid and accurate risk stratification at the ED is essential to optimize patient care and improving outcomes. Various prediction models, ranging from models based on a set of laboratory tests and vital signs (e.g. MEWS, SOFA, RISE UP) to emerging models based on ML have been proposed to aid in this stratification. Unfortunately, most of these models lack external validation, precision, or generalizability to the heterogeneous ED population [13]. In line with the emerging trend of ML technology based prediction models, we developed the RISK^INDEX^, a ML based prediction model that uses demographic characteristics (i.e. age and sex) and the results of laboratory tests available within the first two hours of ED presentation to create an individualized, precise and rapid risk estimation of 31-day mortality. The RISK^INDEX^ has externally been validated in three other hospitals, showing high prognostic accuracy (AUC ranging from 0.88 to 0.98) [16]. To the best of our knowledge, the MARS-ED study is the first prospective, randomized study to investigate the clinical impact of a ML based prediction model in the ED.

We aim to investigate the clinical impact of the RISK^INDEX^ by reviewing the number of changes in the medical treatment plan made as a result of presentation of the RISK^INDEX^ to the ED physician. In addition, we aim to prospectively investigate both the prognostic accuracy of the RISK^INDEX^ and the clinical impact of such a predictive model, and compare the RISK^INDEX^ to the physician’s clinical intuition. By randomizing the patients into an intervention group and a control group, we also aim to assess the clinical impact by comparing the treatment plan in both groups. The ultimate goal of this pilot study is to provide future studies with meaningful estimates of clinical impact of a ML based clinical prediction model.

While a limited number of retrospective studies have described attempts to use ML technology for risk stratification in the ED, none of these studies have been prospectively validated in a randomized setting [14, 15, 26, 27]. In other studies, ML based prediction models were developed with prognostic accuracies similar to our study (AUC of 0.96 and 0.93) [26, 27]. However, to our knowledge, no prospective, randomized trials validating these models have been conducted.

### Limitations

The MARS-ED study is a pilot study that aims to provide future studies with meaningful estimates of clinical impact of prediction models. The MARS-ED study takes place in a single center in the Netherlands, which may limit the generalizability of the results to other EDs, both in the Netherlands and in other countries with different organization of acute care. However, the generalizability of the results of our study is enhanced because we are performing a broadly designed trial in which all patients who enter the ED for assessment and treatment by an internal medicine specialist can be included, and in which there is also a delayed consent pathway to include patients who are temporarily unable to provide informed consent. Furthermore, a recent multicenter validation study showed that the RISK^INDEX^ can be adapted to each medical center’s population [16]. In that study in four EDs, the RISK^INDEX^ showed very high discriminatory performance (AUC ranging from 0.88 to 0.98), indicating that the RISK^INDEX^ is applicable despite local differences in patient demographics.

Another challenge in this study, which takes place in a crowded ED, is that patients have little time to consider their consent on participation since the RISK^INDEX^ has to be presented within the time frame of the ED stay and just before making definite clinical decisions in order to investigate changes of the treatment plan after presentation of the RISK^INDEX^. In addition, ED patients are sometimes not capable of giving informed consent, due to unconsciousness, a state of delirium or mental shock. We try to overcome these challenges by introducing a deferred consent procedure, in order to prevent inclusion bias and improve generalizability of the results. Furthermore, we have created a study team to include patients, and to monitor and solve logistical issues. A member of the study team will be present in the ED during office hours and some evening shifts, when the most patients visit the ED. However, it is possible that due to crowding and absence of the study team, there is not enough time to include patients which may lead to inclusion bias. In order to address any inclusion bias, we will compare patient characteristics and outcomes in our study sample to those of non-included ED patients during the study period.

Lastly, a challenge when prospectively validating prediction models is that the outcome may be influenced by the intervention. Presenting a high RISK^INDEX^ may alter the physician’s treatment plan and therefore influence the outcome (mortality). Consequently, the prognostic accuracy of the RISK^INDEX^, which is measured as the accurate prediction of mortality, may be underestimated. We aim to overcome this challenge by creating an intervention group, where physicians are presented with the RISK^INDEX^, and a control group in order to investigate the clinical impact of the RISK^INDEX^.

## Conclusion

In summary, the MARS-ED study is a randomized, open-label, non-inferiority pilot clinical trial. The aim of this study is to investigate the prognostic accuracy and the clinical impact of a ML technology based prediction model, which, if encouraging, will allow us to optimize patient care in the future.

### Electronic Supplementary Material

Below is the link to the electronic supplementary material.

Supplementary Material 1

## Data Availability

The data used during this study are available from the corresponding author on reasonable request.

## Abbreviations

ALAT: Alanine transaminase
ANCOVA: Analysis of covariance
ASAT: Aspartate transaminase
AUC: Area under the curve
BP: Blood pressure
BUN: Blood urea nitrogen
CBC: Complete blood count
CI: Confidence interval
CK: Creatine kinase
CONSORT: Consorted Standards of Reporting Trials
CRP: C-reactive protein
CTCM: Clinical Trial Center Maastricht
ED: Emergency department
GCS: Glasgow coma scale
HR: Heart rate
ICD-10: International classification of diseases 10
ICU: Intensive care unit
MARS-ED: Machine learning for risk stratification in the emergency department
MC: Medical Center
METC: Medical ethical committee
MEWS: Modified early warning score
ML: Machine learning
MUMC +: Maastricht University Medical Center +
RCT: Randomized controlled trial
RISE: UP Risk stratification in the ED in acutely ill older patients
RR: Respiratory rate
SOFA: Sepsis-related organ failure
SPIRIT-AI: Standard Protocol Items:Recommendations for Interventional Trials– Artificial Intelligence
