# Supplementary material for: Machine learning for risk stratification in the emergency department (MARS-ED) study protocol for a randomized controlled pilot trial on the implementation of a prediction model based on machine learning technology predicting 31-day mortality in the emergency department
Source: Scand J Trauma Resusc Emerg Med. 2024 Jan 23;32:5. doi: 10.1186/s13049-024-01177-2 (PMC10804603; doi:10.1186/s13049-024-01177-2)
Supplement: Supplementary file 1 — Supplementary Material 1 [file 13049_2024_1177_MOESM1_ESM.docx]

**Supplementary Table 1**. CONSORT 2010, checklist of items that should be included in reports of randomized trials.

|  | Item No | Recommendation | Page No |
| --- | --- | --- | --- |
| **Title and abstract** | 1 | *(a)* Identification as a randomized trial in the title | 1 |
|  |  | *(b)* Structured summary of trial design, methods, results, and conclusions. | 2 |
| Introduction | | | |
| Background | 2 | *(a)* Scientific background and explanation of rationale | 3-4 |
| Objectives |  | *(b)* Specific objectives or hypotheses | 3-4 |
| Methods | | | |
| Study design | 3 | *(a)* Description of trial design, including allocation ratio | 4 |
|  |  | *(b)* Important changes to methods after trial commencement | N.A. |
| Participants | 4 | *(a)* Eligibility criteria for participants | 6 |
|  |  | *(b)* Settings and locations where the data were collected | 5 |
| Interventions | 5 | The interventions for each group with sufficient details to allow replication, including how and when they were actually administered. | 6-8 |
| Outcomes | 6 | (*a*) Completely defined pre-specified primary and secondary outcome measures, including how and when they were assessed | 8-9 |
|  |  | *(b)* Any changes to trial outcomes after the trial commenced | N.A. |
| Sample size | 7 | *(a)* How sample size was determined | 10 |
|  |  | *(b)* When applicable, explanation of any interim analyses | N.A. |
| Randomization | 8 | *(a)* Method used to generate the random allocation sequence | 8 |
|  |  | *(b)* Type of randomization; details of any restriction | 8 |
| Allocation | 9 | Mechanism used to implement the random allocation sequence, describing any steps taken to conceal the sequence until interventions were assigned | 8 |
| Implementation | 10 | Who generated the random allocation sequence, who enrolled participants, and who assigned participants to interventions | 8 |
| Blinding | 11 | *(a)* If done, who was blinded after assignment to interventions | N.A. |
|  |  | *(b)* If relevant, description of the similarity of interventions | N.A. |
| Statistical methods | 12 | (*a*) Statistical methods used to compare groups for primary and secondary outcomes | 10-11 |
|  |  | (*b*) Methods for additional analyses, such as subgroup analyses and adjusted analyses | 10-11 |
|  |  | Continued on the next page |  |

| Results | | | |
| --- | --- | --- | --- |
| Participants | 13 | *(a)* For each group, the numbers of participants who were randomly assigned, received intended treatment, and were analyzed for the primary outcome | N.A. |
|  |  | *(b)* For each group, losses and exclusions after randomization | N.A. |
| Recruitment | 14 | *(a)* Dates defining the periods of recruitment and follow-up | 5, 8 |
|  |  | *(b)* Why the trial ended or was stopped | N.A. |
| Baseline data | 15 | Table showing baseline demographic/clinical characteristics of each group | N.A. |
| Numbers analyzed | 16 | For each group, number of participants (denominator) included in each analysis and whether the analysis was by original assigned groups | N.A. |
| Outcomes and estimation | 17 | *(a)* For each primary and secondary outcome, results for each group, and the estimated effect size and its precision (95% confidence interval) | N.A. |
|  |  | *(b)* For binary outcomes, present both absolute and relative effect size | N.A. |
| Ancillary analyses | 18 | Results of any other analyses performed, including subgroup analyses and adjusted analyses, distinguishing pre-specified from exploratory | N.A. |
| Harms | 19 | All important harms or unintended effects in each group | N.A. |
| Discussion | | | |
| Limitations | 20 | Trial limitations, addressing sources of potential bias, imprecision, and, if relevant, multiplicity of analyses | 12-13 |
| Generalizability | 21 | Generalizability of the trial findings | N.A. |
| Interpretation | 22 | Interpretation consistent with results, balancing benefits and harms, and considering other relevant evidence | N.A. |
| Other information | | | |
| Registration | 23 | Registration number and name of trial registry | 2, 5 |
| Protocol | 24 | Where the full trial protocol can be assessed, if available | N.A. |
| Funding | 25 | Sources of funding and other support, role of funders | 15 |

**Supplementary Figure 1**. Overview of the patient’s timeline in the MARS-ED study.


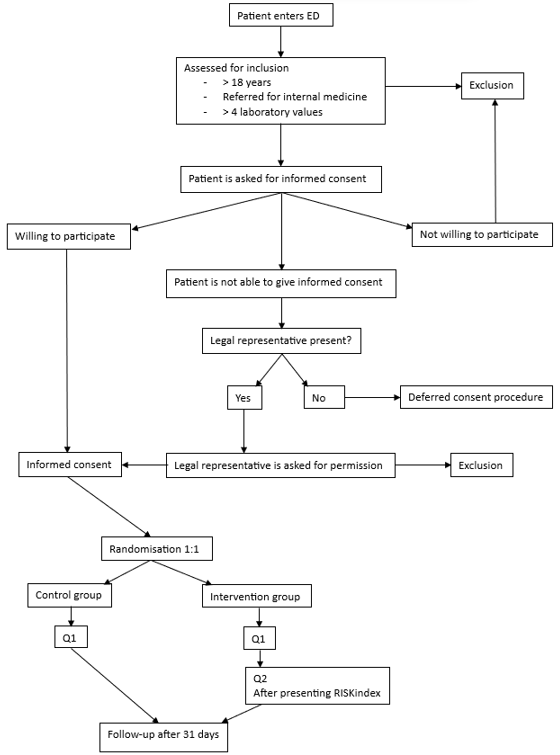


**Supplementary Data**. Additional informed on the RISK^INDEX^.

**Explanation and mechanism of the RISK^INDEX^ prediction model**

The RISK^INDEX^ was developed to support the identification of high and low risk emergency department (ED) patients shortly after admission and the clinical decision making in the ED ^16^. The RISK^INDEX^ uses existing data from the ED. More specifically, it uses basic patient characteristics (age and sex) and the results from laboratory tests. All results available within the first two hours of the ED visit will be used, with the exception of very rarely ordered laboratory tests (prevalence of less than 0.01%).

In order to develop this prediction model, basic patient characteristics and laboratory test results from approximately 50,000 ED patients from three Dutch hospital from the period from 2008 to 2013 were used ^16^. These patient characteristics and laboratory test results were entered into a computer to create a decision tree (Figure 1). This structure was repetitively elaborated by the computer by comparing values to distinguish between living and deceased patients at 31 days. As soon as the best possible distinction was reached, the algorithm stopped developing the model and a fixed structure was created. This method is known as a random forest model. A risk score can be generated for future patients using this automated decision tree. This structure is set and will not be adapted during the study period.

**Supplemental Figure 2.** Schematic view of a decision tree and an example of the current RISK^INDEX^ prediction model.


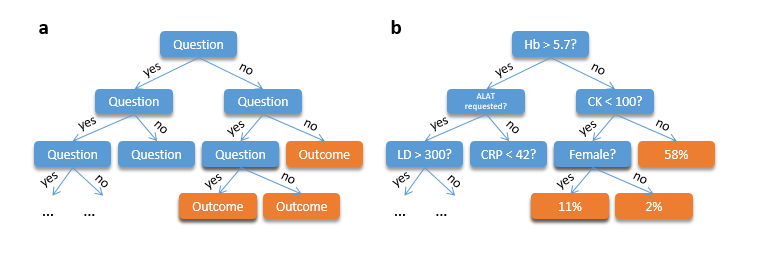


1. Schematic view of a decision tree. At every junction, a yes or no Question is asked. This process is repeated until a certain Outcome is reached (orange squares).
2. Schematic view of the current RISK^INDEX^ prediction model. The model uses laboratory test results (Hb, ALAT, CK, LD, CRP) and patient characteristics (age, sex) to generate a certain outcome (orange squares).

**Precision and validity**

The RISK^INDEX^ model was initially developed for ED patients with sepsis, showing an area under the receiver operating characteristic curve (AUC) of 0.85 (95% CI: 0.78-0.92) ^28^. Subsequently, the model was further developed to fit all medical ED patient and showed an AUC of 0.94 (95% CI: 0.94-0.95). These findings were validated in three other Dutch hospital with comparable diagnostic accuracy (AUC ranging from 0.88 to 0.98) ^16^. Lastly, the model was externally validated in ED patients with COVID-19 with an AUC of 0.80 (95% CI: 0.76-0.85) ^29^.
